# Adjuvant therapy for HER2 positive pT1a-b pN0 breast cancer: A single center cohort study

**DOI:** 10.1097/MD.0000000000029371

**Published:** 2022-06-24

**Authors:** Xuan Yang, Chong Xiao Qu

**Affiliations:** a Department of Breast Surgery, Shanxi Provincial People's Hospital, Taiyuan Shanxi China; b Department of Pathology, Shanxi Provincial People's Hospital, Taiyuan Shanxi China.

**Keywords:** breast cancer, HER-2, node negative, small tumor, systemic therapy, trastuzumab

## Abstract

Deciding if patients with small (≤1 cm), node-negative, human epidermal growth factor receptor 2 (HER2) positive breast cancer should receive adjuvant systemic therapy remains a challenge. No randomized clinical trials have examined the efficacy of trastuzumab in this setting. This prospective observational study aimed to investigate the choice of adjuvant systemic therapy in clinical practice in China.

We prospectively collected data from patients with HER-2 positive breast cancer (less than 1 cm and node negative) patients who underwent breast cancer surgery at Shanxi Provincial People's Hospital Breast Center from January 1, 2017 to December 31, 2019, and retrospectively investigated the association between baseline clinicopathological features and treatment strategy, cardiotoxicity, and disease outcome.

Of 168 eligible patients, 102 (60.7%) received adjuvant systemic therapy with trastuzumab (AST+T), 47 (28%) received adjuvant systemic therapy without trastuzumab (AST) and 19 (11.3%) did not receive adjuvant systemic therapy. Multivariate logistic regression analysis demonstrated that age, tumor size and hormone receptor status were significantly associated with treatment choice. Three-year invasive disease-free survival probability was 100%, 97.9% and 89.5% with AST+T, AST, and no therapy, respectively (*P* < .001).

The majority of patients (60.7%) with pT1a-b pN0 HER2 positive breast cancer received adjuvant systemic therapy with trastuzumab, whereas only 11.3% did not receive any adjuvant systemic therapy. Tumor size, age and hormone receptor status influenced treatment choice. The 3-year invasive disease-free survival probability was significantly higher for patients who received adjuvant systemic therapy with trastuzumab compared with those who did not receive adjuvant systemic therapy. Cardiac adverse events were rare.

## 1 Introduction

The need for adjuvant chemotherapy and 1 year of trastuzumab in patients with <1 cm (pT ≤1 cm) HER2+ node negative breast cancer is still unclear. Many retrospective series have demonstrated that HER2 overexpression were associated with worse outcome in untreated T1a/T1bN0 tumors.^[1–3]^ The 5-year recurrence-free survival rates were 77.1% (95% confidence interval [CI], 67%–85%).^[2]^

However, 2 recent studies^[4,5]^ have provided different results. The lower distant recurrences (ranging from 2% to 7%) in untreated patients did not seem to require adjuvant systemic therapy. Guidelines from the National Comprehensive Cancer Network suggest that adjuvant chemotherapy with trastuzumab should be considered in patients with pT1abN0 (or N1mi micrometastases) HER2+ tumors.^[6]^ The datas were drawn from non-randomized retrospective studies. No available randomized controlled trial or level 1 evidence work has evaluated the role of trastuzumab in women with pT1abN0M0, HER2 positive breast tumors. We designed a single center cohort study to identify clinicopathologic features that may influence the choice of adjuvant systemic therapy, assess the outcome and cardiac safety of adjuvant therapy and offer more evidence that may be used in clinical practice.

## 2 Patients and methods

### 2.1 Patients

A retrospective analysis of prospectively collected data of patients with HER2-positive small tumors (less than 1 cm) treated at Shanxi Provincial People's Hospital Breast Center from January 1, 2017 to December 31, 2019 was performed. Patients were included following pathologic confirmation that the breast cancer was ≤1 cm in size and that the patient had node-negative disease. All tissue specimens were examined by hospital pathologists. Histologic grade and biologic features were evaluated based on the invasive component of the tumor. HER2-positivity was defined as 3+ expression on immunohistochemistry (IHC) or fluorescent in situ hybridization amplification. Estrogen receptor (ER) and progesterone receptor status was also evaluated with IHC. According to the 7th staging system of the American Joint Committee on Cancer (AJCC),^[7]^ tumor size was defined as follows: p (Tmic) micro-invasive carcinoma (≤1 mm of invasive tumor), pT1a (0.1–0.5 cm) and pT1b (>0.5–1.0 cm). Tumors were considered hormone receptor positive if estrogen and/or progesterone receptors were expressed by ≥1% of cancer cells on IHC. A Ki67 cut-off value of ≥20% was used to identify tumors with a high proliferation rate.

Patients were classified into one of 3 groups based on their receipt of adjuvant systemic therapy: no adjuvant systemic therapy, adjuvant systemic therapy without trastuzumab and adjuvant systemic therapy with trastuzumab. Adjunct treatment with trastuzumab consisted of weekly paclitaxel (P; 80 mg/m^2^) and trastuzumab (T; 4 mg/kg week 1 followed by 2 mg/ kg for each subsequent week) for 12 weeks, or Docetaxel (75 mg/m^2^) and cyclophosphamide (C; 600 mg/m^2^) and trastuzumab (T; 8 mg/kg week 3 followed by 6 mg/kg week 3) for a total of 4 doses. After the completion of chemotherapy with trastuzumab, trastuzumab (T; 6 mg/kg week 3) monotherapy was continued to complete a full year. Radiation therapy (RT) was mandated following breast conserving surgery, and adjuvant endocrine therapy in patients with estrogen and/or progesterone receptor-positive tumors was started at the end of chemotherapy. Follow-up studies including clinical examination, complete blood chemistry, chest radiograph and liver ultrasound were performed every 6 months over the first 5 years after surgery, and yearly thereafter. Mammography was repeated every 12 months. Patients ≥70 years old or with a history of heart disease received adjuvant chemotherapy plus trastuzumab under multidisciplinary team (MDT) direction. This study was approved by the Ethical Committees of Shanxi Province People's Hospital. All patients provided the informed consents.

### 2.2 Outcomes

Invasive disease-free survival was defined as the time from surgery for the primary breast cancer until the first local, regional or distant invasive recurrence or death from any cause.

Cardiac events, defined as a symptomatic or asymptomatic left ventricular ejection fraction (LVEF) decline from time of diagnosis to the most recent follow up, were recorded. Cardiac monitoring consisted of a physical examination, a cardiac questionnaire, an electrocardiogram and LVEF measurement on echocardiography. Patients enrolled into all 3 study groups received the same cardiac monitoring: at baseline and 3, 6, 12, 18, and 24 months after therapy start, with yearly checks thereafter. Trastuzumab was interrupted if grade 3 to 4 left ventricular systolic dysfunction (LVSD) was diagnosed or if there was a significant yet asymptomatic decline in LVEF. An asymptomatic decline in LVEF was considered significant if one of the following factors was true: a 10% to 15% decrease from a baseline LVEF that was less than 50%, or a decrease greater than or equal to 16%.

### 2.3 Statistical analysis

Descriptive statistics were used to characterize clinicopathologic and treatment characteristics. The percentage of patients who received chemotherapy with or without trastuzumab was calculated via subgroup comparisons. A multiple logistic regression was used to examine associations between clinicopathological features and treatment regimen choice. Kaplan–Meier curves and log-rank tests were used to compare the survival of the different treatment groups. All *P* values were 2 sided and values less than .05 were considered statistically significant. IBM SPSS Statistics 22.0 (Armonk, NY) was used for data analysis.

## 3 Results

### 3.1 Baseline clinical characteristics and choice of systemic adjuvant therapy

**Figure 1:**
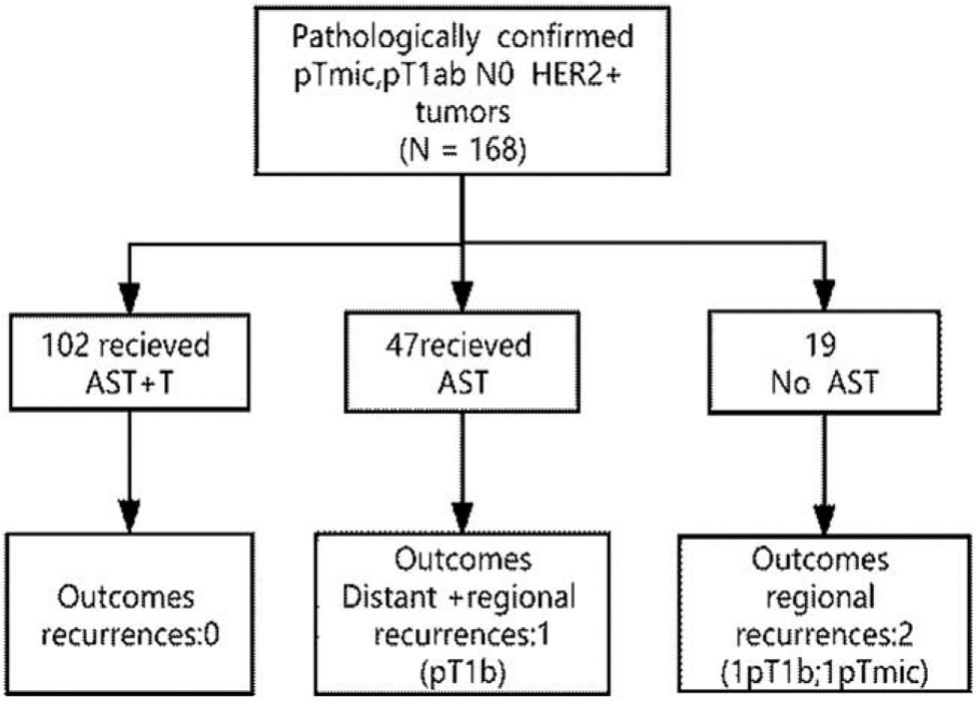
Flow diagram showing selection of patients with pT1abN0 HER2-positive breast cancer.

A total of 168 patients met the inclusion criteria for this study (Fig. 1). Their median age at surgery was 52 years, and 66% were postmenopausal. Tumor size was Tmic for 32 (19%) patients, pT1a for 65 (38.7%) patients and pT1b for 71 (42.3%) patients. The majority of tumors were invasive ductal carcinomas (94.1%) and were moderate (G2, 17%) or high grade (G3, 82%) with a high proliferative index (74%). One hundred fifty eight patients (94%) had pure invasive ductal carcinomas and 94 (56%) were hormone receptor positive. Five tumors (2.9%) had estrogen-receptor positivity of 1% to 10%. HER2 3+ was detected in 151 tumors (89.8%). The remaining 17 patients had equivocal HER2 expression on IHC (2+) but had fluorescent in situ hybridization amplifications between 6 and 20 in 12 cases and between 2.9 and 6.3 in the 5 other cases. Patient characteristics are summarized in Table 1. Local treatments and adjuvant systemic therapies are summarized in Table 2.

**Table 1:** Baseline patient and tumor characteristics.

	All patientsn = 168	No Trastuzumabn = 47	Trastuzumabn = 102	No ASTn = 19	*P* value
Age at diagnosis, n (%)					
≤40 (11.9%)	20 (10.7%)	4 (8.5%)	15 (14.8%)	1 (5.2%)	**<.001**
41–59 (57.7%)	97 (58.9%)	34 (72.3%)	57 (55.9%)	6 (32%)	
60–69 (20.2%)	34 (20.2%)	4 (8.5%)	21 (20.5%)	9 (47%)	
≥ 70 (10.2%)	17 (10.1%)	5 (10.7%)	9 (8.8%)	3 (15.8%)	
Tumor size, n (%)					**<.001**
Tmic (≤1 mm)	32 (19%)	9 (19.1%)	16 (50%)	7 (37%)	
T1a (0.1–0.5 cm)	65 (38.7%)	23 (48.9%)	34 (52%)	8 (42%)	
T1b (0.5–1.0 cm)	71 (42.3%)	15 (32%)	52 (73%)	4 (21%)	
Histology, n (%)					**.258**
Ductal	158 (94.1%)	44 (94%)	96 (94.1%)	18 (94.7%)	
Other, including mixed	10 (5.9%)	3 (6%)	6 (5.9%)	1 (5.3%)	
Grade, n (%)					**.109**
1	2 (1.2%)	1 (2%)	1 (1%)		
2	92 (54.8%)	31 (66%)	52 (51%)	9 (47.4%)	
3	74 (44%)	15 (32%)	49 (48%)	10 (52.6%)	
LVI, n (%)					**.521**
Present	48 (28.6%)	15 (32%)	28 (27.5%)	5 (26%)	
Absent	120 (71.4%)	32 (68%)	74 (72.5%)	14 (74%)	
ER status, n (%)					**.017**
Positive	94 (56%)	30 (63.8%)	58 (63%)	6 (31.6%)	
Negative	74 (44%)	17 (36.2%)	34 (37%)	13 (68.4%)	
Ki-67 index, n (%)					**.916**
<20%	54 (32.1%)	17 (36.2%)	29 (28.4%)	8 (42%)	
≥20%	114 (67.9%)	30 (63.8%)	73 (71.6%)	11 (58%)	

AST = adjuvant system therapy, ER = Estrogen receptor, LVI = Lymphovascular infiltration.

**Table 2:** Local treatment and adjuvant systemic therapies.

	All patientsn = 168	No Trastuzumabn = 47	Trastuzumabn = 102	No ASTn = 19
Surgery, n (%)				
Breast conserving	35 (20.8%)	3 (6.4%)	31 (30.4%)	1 (5.3%)
Mastectomy	133 (79.2%)	44 (93.6%)	71 (69.6%)	18 (94.7%)
SLNB	128 (76.2%)	38 (80.9%)	80 (78.4%)	10 (52.6%)
Axillary dissection	40 (23.8%)	9 (19.1%)	22 (21.6%)	9 (47.4%)
Radiation, n (%)	35 (20.8%)	3 (6.4%)	31 (30.4%)	1 (5.3%)
Hormonal Therapy, n (%)	88 (52.3%)	30 (63.8%)	58 (56.9%)	**0**
OFS+TAM	45 (51%)	12 (40%)	33 (56.9%)	**0**
AI	39 (44%)	16 (53.3%)	23 (40%)	**0**
TAM	4 (5%)	2 (6.67%)	2 (3.4%)	**0**
Chemotherapy, n (%)	135 (80.4%)	36 (76.6%)	99 (97%)	**0**
Paclitaxel for 12 weeks	42 (31%)	14 ((38.9%)	28 (28.3%)	**0**
Docetaxel +C for 4 cycles	93 (69%)	22 (61.1%)	71 (71.7%)	**0**

AI = aromatase inhibitors, AST = adjuvant system therapy, C = cyclophosphamide, OFS = ovarian function inhibition, SLNB = sentinel lymph node biopsy, TAM = tamoxifen.

The majority of patients (102/168; 60.7%) with pT1ab pN0 HER2-positive breast cancer in our single center study received adjuvant systemic therapy with trastuzumab, including 16 pTmic patients (50%), 34 pT1a patients (52%) and 52 pT1b patients (74%). Adjuvant systemic therapy had a positive hormone receptor status, 29 (93.5%) received chemotherapy and endocrine therapy and 2 patients received endocrine therapy only. Premenopausal or perimenopausal patients who were estrogen receptor positive >10% received tamoxifen plus ovarian function inhibition.

Postmenopausal women primarily received aromatase inhibitors. A higher percentage of patients in the trastuzumab cohort received chemotherapy versus the no trastuzumab cohort (97% vs 76%, *P* < .0001). Multivariate logistic regression analysis demonstrated that age, T-stage and hormone receptor status were significantly associated with a different treatment group. Compared with the adjuvant system therapy (AST) + trastuzumab group, older patients had a higher chance of receiving no adjuvant systemic therapy or adjuvant systemic therapy without trastuzumab (odds ratio 9.298, 95% CI 3.263–26.495 and 1.156, 95% CI 0.683–1.957, respectively; *P* < .001). Patients with pT1b tumors were less likely to go without adjuvant systemic therapy or receive adjuvant systemic therapy without trastuzumab (odds ratio 0.066, 95% CI 0.020–0.217, and 0.575, 95% CI 0.326–1.103, respectively; *P* < .0001). Patients with a positive hormone receptor status were less likely to receive no adjuvant systemic therapy (odd ratio 0.264, 95% CI 0.069–1.003), but were more likely to receive adjuvant systemic therapy without trastuzumab (odds ratio 1.535, 95% CI 0.721–3.267; *P* = .032) (Table 3).

**Table 3:** Multivariate analysis of the correlation between clinicopathological characteristics and treatment choice.

Variable	Systemic therapy	*P* value	Odds ratio (95% CI)
HR status (Positive vs Negative)	No AST vsAST + trastuzumabAST vsAST + trastuzumab	.032	0.264 (0.069–1.003)1.535 (0.721–3.267)
Grading (G3 vs G1 & G2)	No AST vsAST + trastuzumabAST vsAST + trastuzumab	.208	2.186 (0.622–7.687)0.699 (0.341–1.433)
LVI (Present vs Absent)	No AST vsAST + trastuzumabAST vsAST + trastuzumab	.208	0.631 (0.153–2.594)1.230 (0.567–2.688)
Ki-67 index (>20% vs <20%)	No AST vsAST + trastuzumabAST vsAST + trastuzumab	.835	0.798 (0.225–2.827)0.825 (0.374–1.731)
Age (as continuous variable)	No AST vsAST + trastuzumabAST vsAST + trastuzumab	.000	9.298 (3.263–26.495)1.156 (0.683–1.957)
Stage (pT1b vs pT1a & pTmic)	No AST vsAST + trastuzumabAST vsAST + trastuzumab	.000	0.066 (0.020–0.217)0.575 (0.326–1.103)

AST = adjuvant system therapy, HR = hormone receptor, LVI = Lymphovascular infiltration.

### 3.2 Breast cancer outcomes

**Figure 2:**
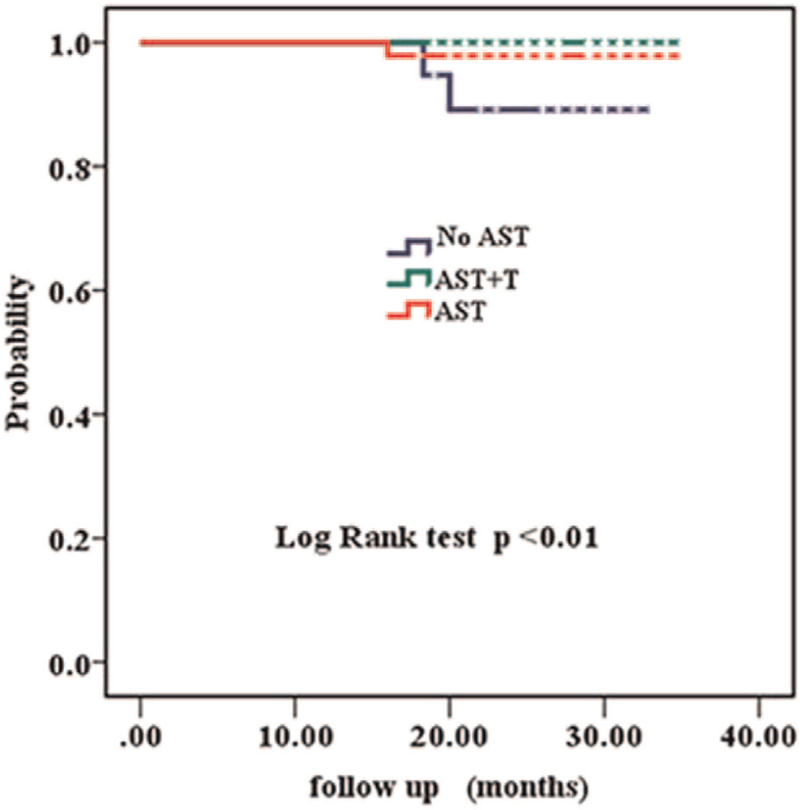
Kaplan–Meier survival curves of all patients.

**Figure 3:**
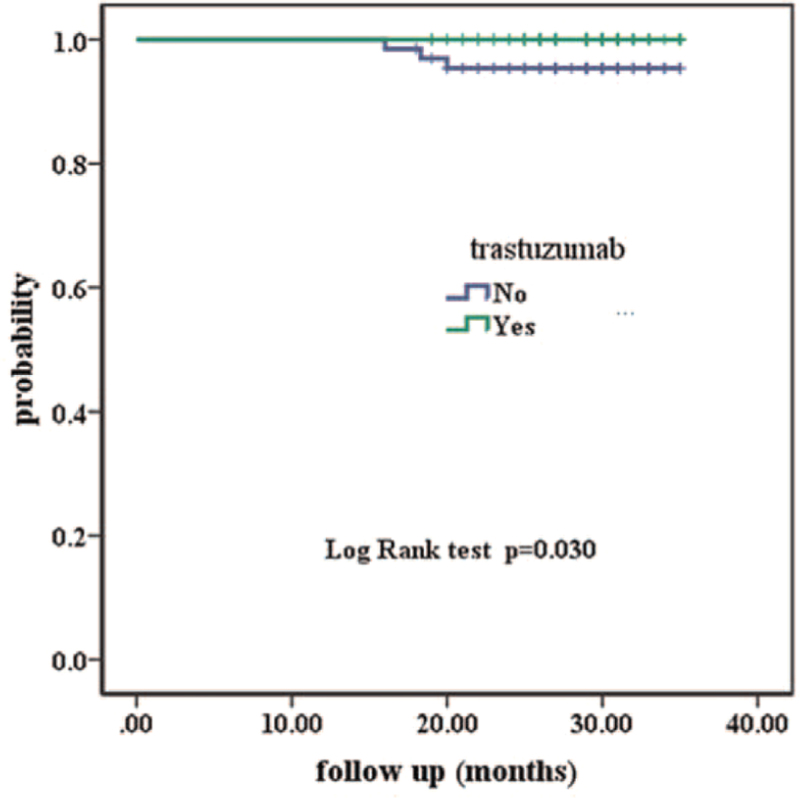
Kaplan–Meier survival curves of patients treated with and without trastuzumab.

**Figure 4:**
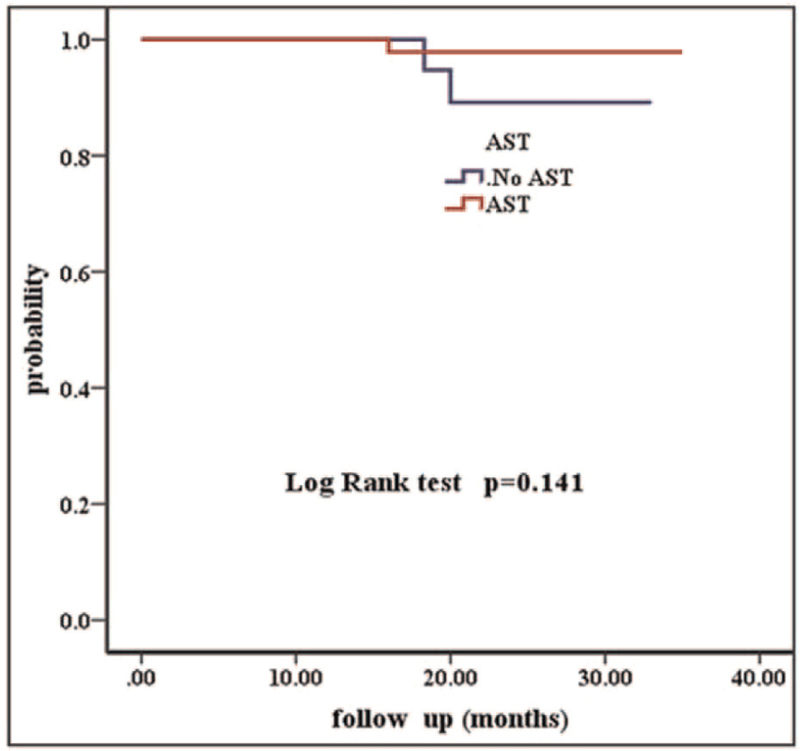
Kaplan–Meier survival curves of patients treated with and without AST.

Over a median follow-up period of 29 (16–35) months, a total of three patients had invasive disease events, yielding an overall 3-year invasive Disease-free survival (IDFS) of 98.2%. Of the 3 recurrences, 2/47 (4.3%) received adjuvant systemic therapy without trastuzumab. One recurrence was in the ipsilateral axilla and the other was in the ipsilateral supraclavicular area and the liver. The other recurrence 1/19 (5.3%) received no systemic therapy, was in the ipsilateral axilla. Patients who received adjuvant systemic therapy with trastuzumab (AST+T) had a 3-year IDFS of 100%. We examined IDFS separately in the AST+T (N = 102) and AST only (N = 47) subgroups compared with untreated patients (3-year IDFS of 100%, 97.9%, vs 89.5% respectively, *P* < .001, Fig. 2). To address in an exploratory way whether trastuzumab impacts adjuvant treatment benefit, we found an IDFS benefit in favor of patients treated with (N = 102) and without trastuzumab (N = 66, 3-year IDFS of 100% vs 95.5%, respectively, *P* = .030, Fig. 3). Among patients receiving AST only, the IDFS difference compared with untreated patients did not reach statistical significance (*P* = .141, Fig. 4). The characteristics of patients who developed a disease recurrence are shown in Table 4.

**Table 4:** Characteristics of patients with a recurrence.

Systemic therapy	Patient	Age	Tumor Size	ER status	Surgery	Pattern of relapse	RFS (months)
No AST	1#	73	pT1b	Positive	Mastectomy+SLNB	regional (HER-2+)	20
No AST	2#	36	pTmic	Negative	Mastectomy+SLNB	regional (HER-2+)	18.3
AST only	3#	40	pT1b	Negative	Mastectomy+SLNB	Regional and distant (HER-2+)	16

AST = adjuvant system therapy, SLNB = Sentinel lymph node biopsy, ER = Estrogen receptor, RFS = relapse free survival; Tmic (≤1 mm);T1b (0.5–1.0 cm).

### 3.3 Cardiac outcomes

Seven (4.2%) patients (95% CI, 1.9%–5.4%) experienced a cardiac event that required the interruption of therapy. One patient in the adjuvant systemic therapy without trastuzumab cohort experienced a significant asymptomatic LVEF decline at the end of cycle 4 of Docetaxel + C. Six patients in the trastuzumab cohort required trastuzumab interruption: 4 had an asymptomatic significant LVEF decline (one each after 6, 9, 10, and 11 months of therapy, respectively) and resumed therapy after a temporary interruption with additional medications; 2 (1.2%, 36 and 40 years of age) developed grade 3 sinus tachycardia without evidence of congestive heart failure and did not resume trastuzumab; one had an arrhythmia at 12 weeks, and another who had an atrial septal defect with mild pulmonary hypertension at baseline developed grade 3 sinus tachycardia 4 months into therapy. The latter two had baseline and measured LVEFs that were normal and >55%, and were medically managed by cardiologists. No Patient developed grade 3 or worse LVSD. Of the 7 patients who developed a cardiac complication, only 3 had 2 cardiovascular risk factors (≥50 years of age and hypertension) and were taking an antihypertensive medication at baseline. Other common adverse events (Occurring in >10% of patients or considered grade 3 adverse events) that occurred during therapy are shown in Table 5.

**Table 5:** Common adverse events that occurred during therapy.

Event	No Trastuzumab n = 66	Trastuzumabn = 102	No Trastuzumabn = 66	Trastuzumabn = 102
Cardiac event	Total, n (%)	Total, n (%)	Grade 3, n (%)	Grade 3, n (%)
Asymptomatic				
LVEF decline	2 (3.0%)	6 (5.9%)	1 (1.5%)	4 (3.9%)
Arrhythmia	1 (1.5%)	3 (2.9%)	0	2 (1.9%)

Other events	Total, n (%)	Total, n (%)	Grade 3, n (%)	Grade 3, n (%)
Neutropenia	9 (13%)	11 (11%)	3 (4.5%)	8 (7.2%)
Fatigue	12 (18%)	19 (19%)	4 (6.0%)	5 (4.9%)
Nausea	19 (29%)	34 (33%)	1 (1.5%)	2 (1.9%)
Neuropathy	9 (15%)	17 (16.6%)	1 (1.5%)	1 (0.9%)
Diarrhea	5 (8%)	13 (12.8%)	2 (3.0%)	3 (2.9%)
Arthralgia	9 (13%)	11 (11%)	1 (1.5%)	1 (0.9%)
Mucositis	4 (5%)	8 (8%)	1 (1.5%)	1 (0.9%)

LVEF = left ventricular ejection fraction.

## 4 Discussion

In this single center study, the majority of patients (102/168; 60.7%) with pT1ab pN0 HER2-positive breast cancer received adjuvant systemic therapy with trastuzumab, including 16 (50%) pTmic patients, 34 (52%) pT1a patients and 52 (74%) pT1b patients. The majority of older patients (64.8%, 24/37, ≥60 years and 52.9%, 9/17 ≥ 70 years) received systemic therapy with trastuzumab, and the majority (97%) of the trastuzumab cohort received chemotherapy. Tumor size, age and hormone receptor status were significantly associated with the administration of adjuvant systemic therapy with trastuzumab on multivariate analysis (Table 1).

This study identified the clinical and pathologic characteristics that may have influenced the decision-making of oncologists in China as to the choice of adjuvant systemic therapeutic strategy for early stage breast cancer. A high number (135, 80.4%)of patients were treated with chemotherapy in our study compared with prior works from other regions. In a French multicenter retrospective cohort study of 356 HER-2 positive pT1abN0M0 tumors, 138 patients (39%) were treated with trastuzumab-based chemotherapy, 29 (8%) with chemotherapy alone and 189 (53%) received neither trastuzumab nor chemotherapy.^[8]^ Retrospective data from a Japanese population-based study ^[9]^ performed from 2004 to 2011 found that the administration rates of chemotherapy and/or trastuzumab stabilized at 50% to 70% for T1b and T1c tumors, and at 20% to 30% for T1a patients. Being ER- was not a predictive marker for pT1ab tumors. Japanese physicians tended to avoid adjuvant chemotherapy for patients with ER+HER2+tumors.

Kolben et al^[10]^ found that the prognosis of N0 tumors <1 cm in size is excellent, especially if they are hazard ratio (HR)-positive. Even in HER2-positive cases there does not seem to be a need for chemotherapy for tumors <0.5 cm. However, if stage pT1b tumors are HER2-positive and HR-negative, chemotherapy may be considered. In our trial, 85 (80%) patients with ER+HER2+ received adjuvant chemotherapy. The higher chemotherapy uses in our study compared with previous studies.^[8–10]^ In 2018 the use of trastuzumab was covered by Medicare in China.

Several patients could not receive the ideal treatment because of economic reasons or their own choice. Chemotherapy without trastuzumab may be an option for these patients. Our population was more likely to be representative of a “real-life” cohort in a developing or economically underdeveloped country. Tumor size seems to have played an important role in treatment choice, as shown by multivariate analysis (*P* < .001) (Table 4). Patients who did not receive any adjuvant systemic therapy had mostly pTmic and pT1a tumors (15/19, 79%; Table 1), whereas patients treated with adjuvant systemic therapy plus trastuzumab were more likely to have pT1b tumors (52/71,73% vs T1a 34/65 54%; Tmic 16/32; 50%; Table 1). Guidelines from the National Comprehensive Cancer Network suggest that adjuvant chemotherapy with trastuzumab should be considered in patients with small, node-negative tumors, including patients with T1bN0 tumors (with N0 denoting no regional lymph-node involvement). Retrospective data from the Japanese National Clinical Database (NCD) showed that systemic treatment improved the outcomes of patients with pT1b disease (breast cancer-specific survival breast-conserving surgery: HR 0.17; 95% CI 0.03–0.95 and OS: HR 0.20; 95% CI 0.06–0.67). However, the treatment of pT1a tumors was not associated with an improved prognosis.^[9]^ Correct estimation of tumor size remains technically challenging. However, tumor size alone did not dictate adjuvant treatment choice. Other bio-pathological and clinical characteristics had an impact on treatment choice, such as age and hormone receptor status. In a Netherlands retrospective study of 3512 patients with stage I HER2-positive breast cancer, 385 (11%) patients had a T1a tumor (including 54 with micro-invasive disease ≤1 mm), and 800 patients (23%) with a T1b tumor had a 5% breast cancer specific survival improvement with systemic treatment in each of the three tumor size subgroups T1a, T1b, and T1c HER2-positive breast cancer.^[11]^ To address in an exploratory way whether trastuzumab impacted adjuvant treatment benefit, we examined IDFS separately in the AST only (N = 47) and AST+T (N = 102) subsets compared with untreated patients. As shown in Fig. 3, patients receiving AST only and AST+T had a similar IDFS, but only in the latter group did a comparison with untreated patients reach statistical significance. There was no difference in IDFS for treated versus untreated patients in the T1a groups (3-year IDFS = 100%). Furthermore, patients with pT1b tumors treated without trastuzumab had worse outcomes (3-year IDFS of 87.5% vs 100% (AST+T), *P* = .008). The difference in IDFS in the Tmic group was not significant (100% vs 93.8%; *P* = .317). Given the small sample sizes compared in these subgroup analyses, their results should be viewed as exploratory only. However, our findings are consistent with those of larger previous reports.^[10–13]^

Nonneville et al ^[8]^ in a multicenter retrospective cohort study examined the outcomes of subcentimeter, node-negative, HER2+ breast cancer and found a significantly higher DFS in patients who received adjuvant trastuzumab-based chemotherapy (HR 0.158 95% CI 0.045–0. 549; *P* = .0037). The benefit of adjuvant chemotherapy ± trastuzumab was independent of tumor size, histologic grade, and hormone receptor status. However, most of the therapeutic benefit seemed to be derived from pT1b tumors. A similar numerical trend was seen in the subset of patients treated with adjuvant chemotherapy only, but it did not reach statistical significance and was likely underpowered to permit any conclusion on the specific impact of trastuzumab on treatment efficacy. Rodrigues et al.^[12]^ in their retrospective multicenter cohort of 276 cases of HER2 + pT1abN0 breast cancer reported a significantly higher DFS in patients who received adjuvant trastuzumab-based chem otherapy. Of the 303 patients enrolled in an observational Italian study,^[13]^ 204 received adjuvant systemic therapy with trastuzumab and 65 received adjuvant systemic therapy without trastuzumab. There was no difference in the 5-year disease-free survival between these treatment groups (95% with trastuzumab vs 94.3% without, adjusted *P* = .621). As the adjuvant systemic therapy without trastuzumab group primarily consisted of patients with hormone receptor positive tumors who received adjuvant endocrine therapy, these results suggest that trastuzumab could be avoided in select cases of hormone positive breast cancer.

In our study; patients ≥60 years of age were more likely to treated with paclitaxel+T, while those <60 years patients were more likely to receive docetaxel cyclophosphamide + T. The APT trial^[14]^ (including 192 patients with pT1a-b pN0 HER2-positive breast cancer, 33.7% patients older than 60 years and 10.1% patients ≥70 years old) demonstrated that paclitaxel for 12 weeks with trastuzumab was well-tolerated, with an incidence of grade 3 to 4 LVSD (congestive heart failure CHF) of only 0.5% (95% CI, 0.1–1.8). The phase II trial by Jones et al ^[15]^ using docetaxel and cyclophosphamide with trastuzumab reported 2 grade 3 cardiac events (0.4%). A borderline low LVEF of less than 55%, an age >50 years, hypertension and a high body mass index are predisposing risk factors for trastuzumab- induced cardiotoxic effects, whereas diabetes, valvular heart disease and coronary artery disease were not statistically influential. Our data were better than that reported in historical studies.^[14,15]^ Over a relatively short follow-up period we found that the 2 regimens were well tolerated (no patient developed grade 3 or worse LVSD). The low incidence of LVEF decline in our study was most likely attributable to the absence of patients who received anthracycline therapy (baseline LVEF ≥55% and MDT by a multidisciplinary team of physicians). Minimal data is available regarding adjuvant trastuzumab treatment choice in older breast cancer patients, especially those with small, node-negative and HER2-positive disease. Our data found that (MDT) elder breast cancer patients do not experience greater treatment toxicity, and that the p a c l i t a x e l + T regimen is well tolerated. In a recent randomized controlled trial by Sawaki et al ^[16]^ that included 275 patients aged 70 to 80 years with HER2-positive early breast cancer, most patients (43.6%) had stage I disease. In these patients the 3-year DFS was 89.5% (95% CI, 82.9%–93.6%) with trastuzumab monotherapy versus 93.8% (95% CI, 87.9%–96.8%) with trastuzumab + chemotherapy (HR, 1.36; 95% CI, 0.72–2.58; *P* = 5.51). Given its lower toxicity and favorable HRQoL profile (health-related quality of life), no patients discontinued trastuzumab treatment because of toxicity. Age >75 years, performance status (PS)1 and ER positivity had a relatively small influence on the effects of chemotherapy. Trastuzumab monotherapy can therefore be considered as an adjuvant therapy option for select older patients who are healthy and have small tumors and good cardiac performance.

In our study only 3 patients in the AST+T group were ≥70 years-old, and , received endocrine therapy with trastuzumab without chemotherapy ;64.8% (24/37) of patients ≥60 years and 35.3% (6/17) ≥70 years received adjuvant chemotherapy with trastuzumab. IDFS 3 years following chemotherapy and trastuzumab was 100%. Trastuzumab with or without Chemotherapy can be used for older patients with HER2 positive early breast cancer. Healthy patients with good performance baseline measurements were able to tolerate standard chemotherapy with trastuzumab, which results in the best prognosis. In the de-escalation ATEMPT trial ^[17]^ the use oftrastuzumab emtansine (T-DM1) was associated with a low rate of recurrence compared with paclitaxel + trastuzumab, but did not meet the pre-planned relative reduction in toxicity. Biomarkers will be important to selecting a suitable population that can achieve an optimal benefit from chemotherapy compared with trastuzumab. The results from other de-escalation studies are pending.

Our study has several limitations, which primarily include its non-randomized design with the potential for selection bias and the lack of a standardized treatment strategy, which means that treated and untreated patients have different prognostic features. The small sample size did not permit a definitive conclusion on the specific impact of trastuzumab on treatment efficacy. However, one of the key strengths of this study is that all patients who were diagnosed with pT1a-b pN0 (including pTmic) HER2-positive breast cancer in our center were evaluated, and there was no selection bias or loss to follow-up.

## 5 Conclusion

In conclusion, in our single center study, the majority of patients (102/168; 60.7%) with pT1a-b pN0 HER2-positive breast cancer received adjuvant systemic therapy with trastuzumab. The main clinical and pathologic characteristics that influenced oncologists in their choice of treatment were tumor size, hormone receptor status and age. After a median 29 months of follow up there was an IDFS benefit in favor of patients treated with trastuzumab compared with untreated patients. Our study adds further data to the growing body of evidence that adjuvant trastuzumab treatment may be beneficial in the treatment of pT1a-b pN0 tumors (including pTmic). Consistent with most of the international guidelines, trastuzumab-based adjuvant chemotherapy should be considered in the treatment of subcentimetric node-negative HER2+ breast cancer. The nature of the cytotoxics that should be administered with trastuzumab remains of debate. De-escalation anthracycline-free and chemotherapy-free regimens for select patients (low-risk or older patients) may be an option. A biomarker analysis is necessary to identify the group of patients who may optimally benefit from combination therapy. At present this remains part of the individualized decision-making by physicians and patients.

## Acknowledgments

We thank all of the patients and physicians for their participation, and the study coordinators for their assistance. We appreciate the financial support by the Shanxi Provincial Health and Family Planning Commission (Grant number: 201601019). All financial sponsors had no role in the design of the study or the collection, analysis or interpretation of study data.

## Author contributions

**Data curation:** Chongxiao qu.

**Validation:** Chongxiao qu.
